# 4-dimensional, multiphase, steady-state imaging with contrast enhancement (MUSIC) in the heart; a feasibility study in children

**DOI:** 10.1186/1532-429X-17-S1-Q131

**Published:** 2015-02-03

**Authors:** Fei Han, Stanislas Rapacchi, Sarah N Khan, Ihab Ayad, Isidro Salusky, Adam Plotnik, Simon Gabriel, Paul J Finn, Peng Hu

**Affiliations:** 1 Radiological Sciences, David Geffen School of Medicine at UCLA, Los Angeles, CA, USA; 2 Department of Bioengineering, University of California, Los Angeles, Los Angeles, CA, USA; 3 Department of Anesthesiology, David Geffen School of Medicine at UCLA, Los Angeles, CA, USA; 4 Department of Pediatrics, David Geffen School of Medicine at UCLA, Los Angeles, CA, USA; 5 Biomedical Physics Inter-Departmental Graduate Program, University of California, Los Angeles, Los Angeles, CA, USA

## Background

Conventional breath-held gadolinium-based contrast-enhanced MR angiography (CE-MRA) provides excellent definition of extra-cardiac anatomy, but does not provide diagnostic image quality for intra-cardiac anatomy because it is not gated to ECG due to time constraints associated with breath-holding and the need to capture the first-pass of gadolinium. To address this issue, we propose a 4D non-breath-held multiphase steady-state imaging sequence (MUSIC) using ferumoxytol, which is an FDA approved iron-oxide particle for treating iron-deficiency anemia, as an intravascular contrast agent and demonstrate its feasibility in children with congenital heart disease (CHD).

## Methods

Eight pediatric CHD patients underwent cardiac MRI under general anesthesia and mechanical ventilation, which is our standard practice for young children who cannot cooperate with breath-holding instructions. The breath-held CE-MRA was first performed under ventilator-controlled breath-hold (VCBH) during the first-pass of a ferumoxytol bolus injection (4 mg-Fe/kg) and repeated 2-3 min later during the delayed-phase. Subsequently, the cardiac-phase-resolved 4D MUSIC acquisition (TR/TE= 2.9/0.9ms; FA=15°; isotropic resolution=0.6-0.9mm, 5-8 cardiac phases, scan time 4-8min, 65-95ms temporal resolution) was performed during the steady-state distribution phase of ferumoxytol without VCBH and the ventilator tubing air pressure signal was fed into the MR scanner for respiratory gating. Standard 2D cardiac cine images were also acquired under VCBH. Subjective image quality scores (1=poor, 2=fair, 3=good, 4=excellent) were visually assessed and the LV volumes were measured based on standard 2D cine and the 4D MUSIC data.

## Results

**Figure 1:**
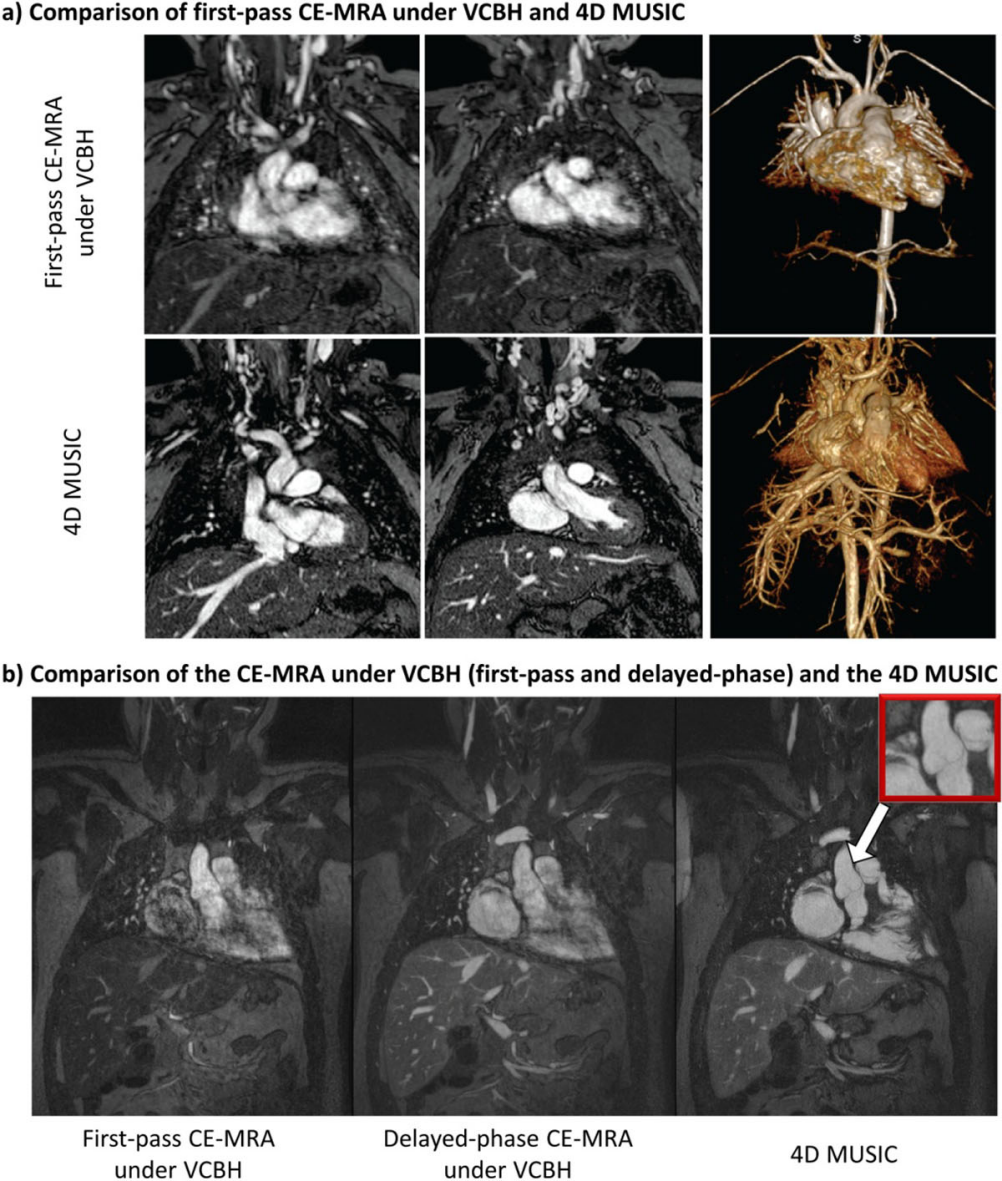
a) Selected 2 slices and 3D rendering of first-pass CE-MRA under VCBH and the 4D MUSIC (phase #3 is chosen out of 5 cardiac phases for display) on a 4 y.o. 10kg boy. The structures in and close to the heart (including the cardiac chambers, coronary arteries, aortic root, main pulmonary artery, and valves) are much better defined using 4D MUSIC. b) Comparison of the first-pass VCBH CE-MRA, delayed phase VCBH CE-MRA, and the proposed 4D MUSIC. The 4D MUSIC image provided excellent definition of cardiac anatomy including the aortic cusps and the aortic valve leaflets (red box), and the trabeculations within the cardiac chambers, while both first-pass and delayed-phase VCBH CE-MRA suffers from significant cardiac motion blurring. Signal intensity of the 4D MUSIC image is uniform across the entire FOV.

**Figure 2:**
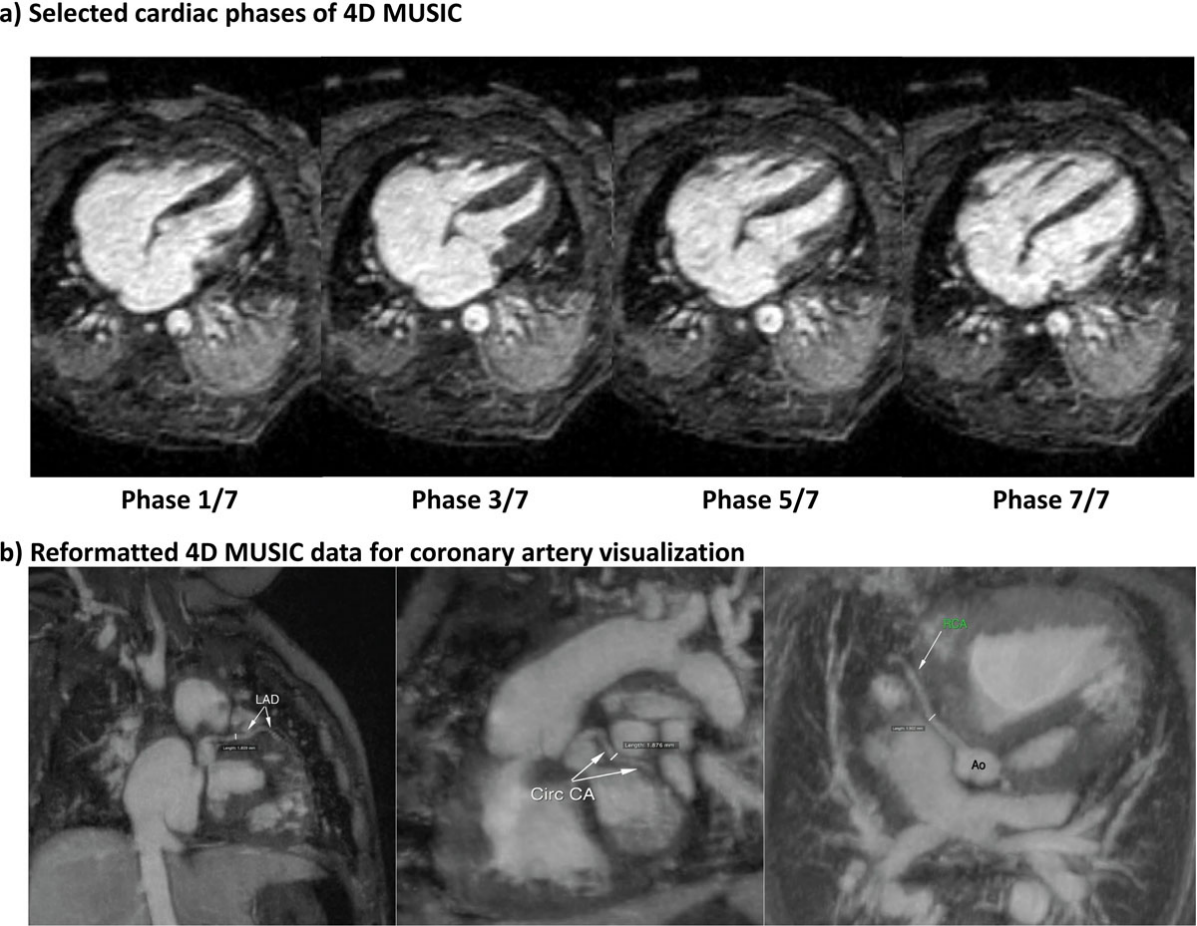
a) Reformatted cardiac four chamber view images on selected cardiac phases (4 out of 7) based on the 4D MUSIC on a 3 d.o. 2kg boy. No 2D cine was acquired on this patient due to concerns of cardiopulmonary inefficiency and clinical assessment of cardiac function and volumes was based on the 4D MUSIC data. b) All three major branches of the coronary artery (LAD: left anterior descending; Circ CA: left circumflex; RCA: right coronary arteries) are clearly visualized by reformatting the 4D MUSIC data acquired in an 8-month-old 7kg boy with complex CHD. The multiphase data have 0.9mm isotropic resolution.

As shown in Fig. 1, compared to VCBH CE-MRA, the 4D MUSIC provides much improved definition of intra-cardiac structures (e.g. cardiac chambers, coronary arteries and the valves) by eliminating cardiac motion blurring. Fig.2a shows reformatted cardiac four-chamber view at select cardiac phases from which the LV volumes could be calculated. In Fig. 2b, all three major branches of the coronary artery are clearly visualized by reformatting the 4D MUSIC acquired on an 8-month-old patient. In 7 out of the 8 patients, the origins and course of the coronary arteries, including 2 anomalous origins were identified based on the 4D MUSIC image. In one patient, the 4D MUSIC approach pre-empted an invasive diagnostic catheterization. 4D MUSIC provided significantly better image scores for intra-cardiac anatomy than the first-pass MRA (3.6±0.5 vs. 2.0±0.4 P<0.05). The LV volume measurements based on 2D cine and 4D MUSIC correlated well (concordance correlation coefficient >0.95).

## Conclusions

Our study represents a potential new paradigm of cardiovascular MRI for young children. The use of ferumoxytol (14 hours of intravascular half-life) eliminates the time constraints of conventional CE-MRA and enables much higher quality 4D dynamic cardiac imaging with sub-millimeter isotropic resolution.

## Funding

NIH (NHLBI) 1R21 HL113427.

